# Physicochemical and Functional Properties of Active Fish Gelatin-Based Edible Films Added with Aloe Vera Gel

**DOI:** 10.3390/foods9091248

**Published:** 2020-09-07

**Authors:** Jorge Trujillo Sánchez, Arantzazu Valdés García, Antonio Martínez-Abad, Francisco Vilaplana, Alfonso Jiménez, María Carmen Garrigós

**Affiliations:** 1 Analytical Chemistry, Nutrition & Food Sciences Department, University of Alicante, P.O. Box 99, 03080 Alicante, Spain; jorgetrujillosanchez@gmail.com (J.T.S.); alfjimenez@ua.es (A.J.); 2 Division of Glycoscience, Department of Chemistry, School of Engineering Sciences in Chemistry, Biotechnology, and Health, KTH Royal Institute of Technology, AlbaNova University Centre, SE-106-91 Stockholm, Sweden; antma@kth.se (A.M.-A.); franvila@kth.se (F.V.)

**Keywords:** active packaging, edible films, antimicrobial activity, fish gelatin, Aloe Vera

## Abstract

Edible films based on the addition of Aloe Vera gel (AV) into fish gelatin (FG) with antimicrobial and functional properties for food packaging applications were proposed in this work. AV showed an amphiphilic nature by infrared spectroscopy, high total phenolics content (TPC), antioxidant activity and thermal stability with an initial degradation temperature of 174 ± 2 °C. Mannose and glucose were quantified as main monosaccharides whereas the linkage composition study confirmed the presence of acemannan as main active polysaccharide. Three different formulations were obtained by the casting technique and the addition of AV contents of 0, 1 and 4 wt.% to FG, showing films with 4 wt.% of AV the best performance. The addition of AV did not significantly affect mechanical and barrier properties to oxygen and water vapour. However, some structural changes were observed by infrared spectroscopy and the obtained glass transition temperature values due to intermolecular interactions that increased the hydrophilicity and solubility of the resulting FG/AV films. A higher thermal stability was observed in films with AV content increasing the initial degradation and oxidation onset temperatures. An antimicrobial activity against *S. aureus* was also observed for FG/AV films. The addition of AV into FG could be proposed as a potential effective material to increase the postharvest quality of packed fruits and vegetables by retarding the microbial growth and extending the shelf-life of these food products.

## 1. Introduction

Fruits and vegetables are perishable food products susceptible to postharvest quality losses during cold storage and shelf-life periods through weight loss, softening, colour changes and microbial contamination [1,2]. According to the Food and Agricultural Organization of the United Nations (FAO), 1300 million tons of food, about a third of what is produced, is wasted each year, including around 45% of all fruit and vegetables [3]. This situation underlines the need to develop new sustainable alternatives to reduce food waste by both increasing the shelf-life of food products and also by finding sustainable routes to reutilize food by-products and waste streams. Active packaging has emerged as a promising strategy to increase postharvest quality of fresh food products to contribute to the circular economy concept by reducing food waste generation and avoiding the excessive use of chemicals as additives into packaging.

Fish gelatin (FG) has been underlined as a potential protein bio-polymer to replace non-degradable materials due to its film-forming capacity, transparency, flexibility, biodegradability, availability and good barrier properties to UV radiation, oxygen, carbon dioxide and volatile compounds [4,5,6]. Also, the ability of FG to form intermolecular interactions due to the non-polar and polar amino acid components linked to its amphiphilic character has allowed the development of effective active edible films [7,8,9]. Within the wide range of substances used as active additives, natural products obtained from plants are gaining attention because of consumers’ rejection to antioxidants from synthetic origin based on potential toxicity and possible carcinogenic effects [10]. In the last decade, the antioxidant, antimicrobial, antidiabetic, hepatoprotective, anticarcinogenic and antihyperlipidemic effects of Aloe Vera (AV) gel extract have been extensively proved for food packaging applications [11,12,13]. These properties are a consequence of the high content on unique polysaccharides (e.g., glucomannan, acemannan), phenolic compounds (flavonoids and anthraquinones), organic acids and vitamins (B_1_, B_2_, etc.) present in AV [14,15]. Recently, AV has been used as a functional additive in edible matrices to maintain the postharvest quality of some fruits such as mango [16], tomato [17], strawberries [18] and blueberries [19]. Also, edible films based on alginate and AV have demonstrated the potential of this additive to form homogeneous films with good transparency to be used in different fields [20].

Up to now, the potential addition of AV into fish gelatin matrices has not been widely explored. Sui Chin et al. [21] proved the antioxidant properties of fish gelatin films added with Aloe Vera gel without affecting the colour, thickness and surface microstructure of the developed films. Radi et al. [22] proved that gelatin-based coatings incorporated with Aloe Vera and green tea extracts successfully retarded the microbial growth and extended the shelf-life of fresh-cut oranges during cold storage. However, further studies are required to determine the potential antimicrobial properties of FG-based films incorporating AV to prevent the proliferation of pathogenic bacteria in food products; as well as to evaluate the effect of AV addition on the FG structure to explore the potential application of FG/AV edible films. Thus, the purposes of this work include: (a) the characterization of AV in terms of polysaccharide composition, structural, antioxidant and thermal properties; (b) the successful development and full physicochemical and functional characterization of FG-based edible films added with different contents of Aloe Vera gel (0, 1 and 4 wt.%); (c) the study of the antimicrobial capacity of the developed active films for food packaging applications.

## 2. Materials and Methods

### 2.1. Materials

Cold water fish skin gelatin (G2963A) for food applications was obtained from Lapi Gelatin (Empoli, Italy) containing 85–90 wt.% protein, 11 wt.% of moisture, 1–2 wt.% mineral salts and less than 1 wt.% lipids, without the presence of preservatives or other additives. According to the supplier specifications, the Bloom gel strength ranges from 230 to 270 g with an amino acid (AA) composition of 23.7 wt.% glycine, 12.6 wt.% proline, 12.3 wt.% hydroxyproline and 12.1 wt.% arginine as major components, followed by 6.0 wt.% alanine, 4.9 wt.% glutamic acid, 4.1 wt.% serine, 3.7 wt.% threonine, 3.3 wt.% lysine, 3.1 wt.% phenylalanine, 2.8 wt.% leucine, 2.5 wt.% methionine, 2.4 wt.% aspartic acid and 2.0 wt.% valine, among other minor AAs. Glycerol (analytical grade) was obtained from Panreac (Barcelona, Spain) and it was used as plasticiser. Aloe Vera leaves (*Aloe barbadensis* Miller) with 70–90 cm on average height and 8 cm thickness were kindly supplied by Aloe Vera Las Coronas (Carmona, Spain). AV leaves were washed with cold distilled water to clean the surface and dried during 12 h at room temperature before further treatment.

### 2.2. Preparation of Aloe Vera Inner Gel

For AV gel extraction, a conventional industrial protocol was followed [23,24]. In brief, 2.5 cm of the white part of the leaf base, 5–10 cm of the leaf top and the sharp spines along the margins were removed by using a knife. Then, the pulp was manually separated from the inner fillet. This pulp was subjected to a grinding process in a mill. Samples were prepared by using the Thermomix TM5 (Vorwerk, Thermomix, Madrid, Spain) food processor during 5 s under stirring at 10.000 rpm to obtain the inner gel juice. The grinding process was repeated twice in a discontinuous process in order to avoid heating the gel. Then, it was further centrifuged at 5000 rpm for 30 min to remove the suspended solids. Finally, the colourless supernatant was filtrated under vacuum, freeze-dried (Alpha LDplus Entry Freeze Dryer Package, Chatswood, Australia) and stored at −4 °C. The obtained AV dry extract was used as active additive throughout this study.

### 2.3. Preparation of Edible Films

Edible films were prepared by the casting technique. Firstly, AV was diluted in 20 mL of distilled water at room temperature under magnetic stirring (100 rpm) for 10 min. Then, FG (8 wt.%) was dispersed in the AV/water solution at 35 ± 2 °C and 100 rpm for 20 min. Glycerol (25 wt.%) was further added and dispersed for 10 min under the same conditions. To avoid the presence of bubbles, film forming dispersions were sonicated for 30 min and finally dried in Petri dishes at 50% relative humidity (RH) and 23 ± 1 °C in a climate chamber (Dycometal, Barcelona, Spain) for 48 h. Three different formulations were obtained by adding different AV contents to FG (0, 1 and 4 wt.%). The obtained edible films were named as FG (control), FG/AV1 and FG/AV4; where the number corresponds to the added AV content (1 and 4 wt.%, respectively).

### 2.4. Characterization of Aloe Vera Inner Gel

#### 2.4.1. Carbohydrate Composition Study

The sugar composition of AV extracts was determined after performing trifluoroacetic acid (TFA) and sulfuric hydrolysis, respectively. For TFA hydrolysis, freeze-dried samples (1 mg) were incubated with 1 mL of 2 mol L^−1^ TFA for 3 h at 120 °C. Samples were then dried under a stream of air and redissolved in water. Sulfuric hydrolysis was performed by adding 250 µL of 72 wt.% sulfuric acid to approx. 4 mg of sample and incubating the mixture at room temperature for 3 h. Then, deionized water was added to dilute the solution to approx. 1.2–1.3 mol L^−1^ sulphuric acid and the tubes were further incubated at 100 °C for 3 h. The monosaccharides were analysed using high performance anion exchange chromatography with pulsed amperometric detection (HPAEC-PAD) with an ICS-3000 system (Dionex) equipped with a CarboPac PA1 column (4 × 250 mm, Dionex). Inositol was added to all samples as an internal standard prior to hydrolysis. For glycosidic linkage analysis, freeze-dried samples (5 mg) were carboxyl reduced to label the uronic acids present in the fractions and transformed them into partially methylated alditol acetates (PMAAs), as it was previously described [25]. PMAAs were separated and analysed by gas chromatography (HP-6890, Agilent Technologies, Palo Alto, CA, USA) coupled to an electron-impact mass spectrometer (HP-5973, Agilent Technologies) on a SP-2380 capillary column (30 m × 0.25 mm i.d.; Sigma-Aldrich, Madrid, Spain) with a temperature program increasing from 160 °C to 210 °C at a heating rate of 1 °C min^−1^. The mass spectra of the fragments obtained from the PMAAs were compared with those of reference polysaccharide derivatives and with available literature data [26]. Quantification was based on the effective carbon response of each compound. All sugar composition and linkage analysis experiments were carried out in triplicate.

#### 2.4.2. Structural Characterization by ATR-FTIR

AV (2.00 ± 0.01 mg) was directly placed on a Golden Gate single reflection diamond ATR accessory (incident angle of 45°) to study the structural composition of the active additive by using a Bruker Analitik IFS 66 FTIR spectrometer (Ettlingen, Germany) equipped with a DTGS KBr detector. Absorbances were recorded from 4000–500 cm^−1^, using 64 scans and 4 cm^−1^ resolution. Spectra were corrected against the background spectrum of air. Three replicates were obtained for each sample.

#### 2.4.3. Thermal Characterization

The thermal stability of AV (7.0 ± 0.1 mg) was analysed in a TGA/SDTA 851 Mettler Toledo (Schwarzenbach, Switzerland) thermal analyser from 25 to 800 °C at 5 °C min^−1^ under N_2_ atmosphere (50 mL min^−1^). Thermal properties of AV were determined using a differential scanning calorimeter (DSC, TA DSC Q-2000 instrument, New Castle, DE, USA) according to Pereira et al. [20]. Analyses were performed in triplicate.

#### 2.4.4. Total Phenolic Content

The polysaccharides present in AV were precipitated, as described by Lucini et al. [27] prior to analysis, in order to avoid interferences during the quantification of the total phenolic content (TPC) and the determination of the antioxidant activity. The dried supernatant (6.7 wt.% yield from AV, from now on AVR) was re-suspended in 80% ethanol (*v*/*v*) to a final concentration of 255 mg mL^−1^, immediately before analysis. TPC of AVR was determined at 685 nm using a Biomate-3 U*V*/*V*IS spectrophotometer (Thermospectronic, Mobile, AL, USA) as it was described by Ramful et al. [28]. Gallic acid was used as the reference standard and results were expressed as mg of gallic acid equivalents (GAE) per gram of dried AV. The linear range of the calibration curve was 42–200 mg kg^−1^ of gallic acid with an acceptable linearity (R^2^ = 0.9998). Analyses were performed in triplicate.

#### 2.4.5. Antioxidant Activity by DPPH, ABTS and FRAP Methods

The antioxidant activity of AVR was determined by using three complementary spectrophotometric methods with a Biomate-3 UV/VIS spectrophotometer (Thermospectronic, Mobile, AL, USA), in triplicate. The ability of AVR to scavenge the stable free radical DPPH• was measured at 517 nm according to Saini and Saini [29]. Results were expressed as the concentration of substrate (mg extract mL^−1^) that caused 50% loss of the DPPH activity (IC_50_). The linear range of the calibration curve was 0.72–1.33 mg g^−1^ of DPPH• with an acceptable linearity (R^2^ = 0.9992). ABTS^+^ radical scavenging activity was determined at 734 nm according to the protocol-decolorization assay in ethanolic solution described by Re et al. [30]. Gallic acid (2–11 mg kg^−1^) was used as standard with an acceptable linearity (R^2^ = 0.9991). Results were expressed as µg GAE per gram of dried extract. Finally, the capacity of AVR to reduce ferric ions was assessed by the FRAP method according to Ramful et al. [28]. The absorbance was read at 593 nm after 30 min incubation at 37 °C. A calibration curve of gallic acid (2–11 mg kg^−1^) with good linearity (R^2^ = 0.9995) was used. Results were expressed as µg GAE per gram of dried extract.

### 2.5. Characterization of Edible Films

A complete optical, morphological, structural, thermal and mechanical characterization of the obtained edible films was performed in this work. Also, the barrier properties and antimicrobial capacity of the films were studied. All samples were conditioned for 48 h at 50% RH and 23 °C, before analyses.

#### 2.5.1. Thickness, Light Transmittance and Transparency Values

The thickness of films was measured, in triplicate, with a precision of 0.001 mm. Each film sample was measured at five random positions by using a 293 MDC-Lite Digimatic Micrometer (Mitutoyo, Japan).

Absorbance values obtained at ultraviolet (280 nm) and visible (600 nm) radiations using a UV-Vis spectrophotometer (Spectronic BioMate 3, Thermo Electron Corporation, Warwickshire, UK) were used to calculate the transparency values, as it was described by Guerrero et al. [31]. The transparency of the films (%) was calculated as A/L × 100, where A is the absorbance at 280 or 600 nm and L is the film thickness (mm). Three specimens were tested for each composition.

#### 2.5.2. Morphological Characterization

Scanning electron microscopy (SEM) was used to analyse the surface morphology of films (1 × 1 cm^2^) by using a JEOL JSM-840 equipment (Peabody, MA, USA) under an acceleration voltage of 15 kV. Samples were previously coated with gold by using a sputter coater (SCD 004 Balzers, Bal Tec. AG, Furstentum, Lichtenstein, Germany).

#### 2.5.3. Structural Characterization

The film samples structure (1 × 1 cm^2^) was studied by ATR-FTIR as it was described in Section 2.4.2. Three replicates were obtained for each sample.

#### 2.5.4. Thermal Characterization

Thermal properties of films were analysed using the same instrumentation as in Section 2.4.3. In the case of TGA, samples (7.0 ± 0.1 mg) were heated from 25 to 700 °C at 10 °C min^−1^ under N_2_ flow. The initial degradation temperature, T_ini_ (°C), and the temperature of maximum degradation, T_max_ (°C), were determined according to Valdés et al. [32]. For DSC, samples (4.0 ± 0.1 mg) were placed into aluminum pans, sealed and subjected to one heating run (−90 °C to 150 °C) [33,34] at 10 °C min^−1^ under N_2_ atmosphere (50 mL min^−1^). All analyses were performed in triplicate.

The oxidative stability of the tested AV/FG films was evaluated by means of the oxidation onset temperature (OOT) determined by DSC [35]. Samples (8.0 ± 0.1 mg) were heated from 30 °C to 235 °C at 10 °C min^−1^ under oxygen atmosphere (gas flow 50 mL min^−1^). The OOT value corresponds to the onset temperature of the exotherm observed in the temperature scanning experiment.

#### 2.5.5. Mechanical Properties

Three tensile parameters were obtained from the stress-strain curves following the ISO D82-12 standard (D882-09). Five replicates were performed at ambient temperature using a 3340 Series Single Column System Instron Instrument, LR30K model (Fareham Hants, UK) equipped with a 100 kN load cell with an initial grip separation of 50 mm and a crosshead speed of 2 mm min^−1^.

#### 2.5.6. Barrier Properties

Water vapour permeability (WVP) was determined by using the Desiccant Method (CaCl_2_) in a climate chamber (Dycometal, Barcelona, Spain). Oxygen transmission rate (OTR) tests were carried out with an oxygen permeation analyser (8500 model Systech Instruments, Metrotec S.A, Lezo, Spain) according to Valdés et al. [32]. The solubility of films was determined, in triplicate, as it was previously detailed by Hosseini et al. [33].

#### 2.5.7. Antimicrobial Activity

The agar diffusion method was conducted to assess the antimicrobial activity of the obtained films [36]. *S. aureus* and *S. enterica*, were used as Gram positive and Gram negative, respectively, common bacterial strains related to spoilage in refrigerated foods. 0.1 mL of inoculum with approximately 10^5^ CFU mL^−1^ of the tested bacteria was spread onto Muller Hinton agar plates. The diameter of the inhibition zone (mm) around the film disc (1 × 1 cm^2^) was measured after 24 h of incubation at 37 °C. FG was used as control. Triplicates were performed per each sample.

### 2.6. Statistical Analysis

SPSS commercial software (Version 15.0, Chicago, IL, USA) was used for statistical analysis of experimental data. A one-way analysis of variance (ANOVA) and Tukey’s test with a *p* < 0.05 significance level were applied.

## 3. Results and Discussion

### 3.1. Characterization of Aloe Vera Inner Gel

#### 3.1.1. Carbohydrate and Polysaccharide Analysis

The sugar composition of AV, determined after a two-step sulphuric hydrolysis and TFA hydrolysis, is presented in Figure S1. The lack of major differences between both hydrolysis methods suggests the absence of crystalline cellulose in AV, as TFA hydrolysis would not be able to digest it [37]. Mannose (Man) and glucose (Glc) add to about 95% of all the carbohydrate content, with the rest being mainly minor amounts of galactose (Gal), arabinose (Ara) and xylose (Xyl) (Figure S1). The main carbohydrate units, glucose and mannose, can either form part of the bioactive acemannan polysaccharide, or may be free glucose, also present in AV at relatively high amounts [38]. The complete carbohydrate linkage analysis (Table 1) does not take into account monomeric sugar units, so a simple subtraction of the glucose obtained by both methods contributes to estimate the acemannan (83 wt.%) and free glucose (12 wt.%) contents.

The carbohydrate composition of AV evinces that no extraction or purification steps are actually necessary to obtain a gel with a specific and relatively high level of purity in the bioactive polysaccharide. The linkage analysis data confirmed that the polymer is a linear β-1→4 linked glucomannan. The low glucose to mannose ratio of this polysaccharide (1:7) and partial acetylation of either the C-2 and C-3 or C-6 positions, prompted the coined name “acemannan” [39]. The polymer is considered to be linear, because of a very minor degree of galactosyl substitutions at C-6 (only 0.3%mol of 4,6-Man*p* and 0.3%mol of 4,6-Glc*p*; Table 1) [38,40,41].

The purity of the polysaccharide fraction is again patent in the linkage analysis, with hints to the minor presence of xylan and arabinogalactan. The overall linkage composition of AV matches with previous reported results [42], except for a slight variation in the pectin content. In this sense, the pectin in AV has been ascribed to the cell wall content, which is obviously variable depending on seasonal changes, irrigation or geographic location [43,44].

#### 3.1.2. Structural Study by ATR-FTIR

**Figure 1:**
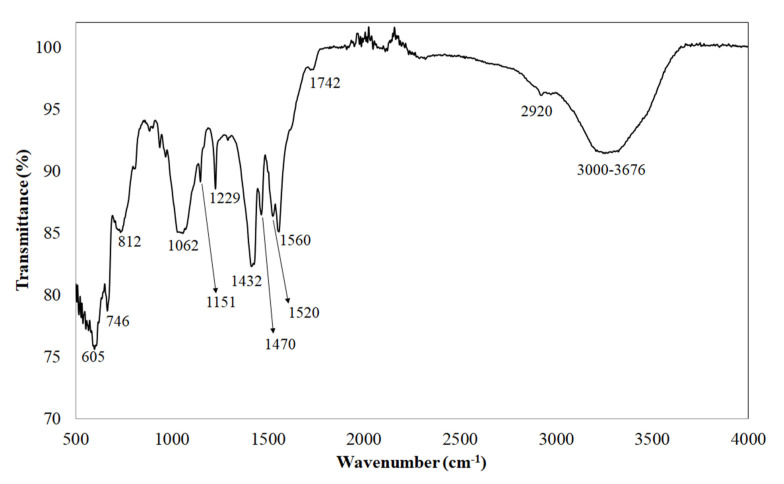
Average ATR-FTIR spectrum of freeze-dried AV extract.

The FTIR analysis of AV revealed the polar nature of the gel as four main functional groups (OH, CO, CH_3_ and COO) were predominant in the AV spectrum (Figure 1). The wide band observed in the range of 3000–3676 cm^−1^ was related to the stretching vibration of phenolic hydroxyl groups such as flavonoids and anthraquinones, among others [15,45,46]. The band observed approximately at 2920 cm^−1^ is characteristic of aliphatic groups (–CH_2_ and –CH_3_) whereas the C=O stretching vibrations of carbonyl groups of polysaccharides and gelatin matrix appeared at 1742 cm^−1^ [46]. The band observed near 1560 cm^−1^ could be related to the N-H bending of the amide II fraction [46]. Other predominant functional groups were the asymmetric COO (band at 1520 cm^−1^), symmetric COO (band at 1470 cm^−1^), CH_3_ stretching vibrations (band at 1432 cm^−1^) and C–O–C stretches of acetyl groups of esters and phenols (band at 1227 cm^−1^) [47]. Regarding the bands related to the presence of polysaccharides, an intense band in the range of 1151–1062 cm^−1^ was associated with the presence of galactose and glucan units [48]; whereas peaks at 812 cm^−1^ and 746 cm^−1^ reflect the characteristic signals of glucose and mannose, respectively [49]. Finally, the absorption peaks around 605 cm^−1^ might be due to C-H bending indicating the presence of polymerized compounds in the AV gel [15].

#### 3.1.3. Thermal Characterization

The calorimetric curve obtained for AV showed three endothermic peaks at 68 ± 3 °C, 170 ± 2 °C and 223 ± 4 °C related to water loss from the polymer hydrophilic groups, polymer depolymerisation and degradation reactions, respectively (Figure S2) [20]. Four main thermal degradation stages were observed by TGA (Figure S3). Firstly, some volatiles from organic acids and residual water were released from the sample at 104 ± 8 °C. Then, the maximum thermal degradation temperature took place at 252 ± 11 °C followed by two additional degradation phases at 445 ± 3 °C and 658 ± 8 °C attributed to the depolymerisation, degradation and carbonization of the AV polysaccharides, respectively [46]. The obtained thermal decomposition profile can be assigned to the presence of acemannan in the gel, which is in accordance with the high purity of the active polysaccharide found in Section 3.1.1 [50]. On the other hand, the T_ini_ value of AV was 174 ± 2 °C, indicating that AV might be thermally stable for casting or coating applications as well as to be processed within a polymer blend at relatively mild conditions. A final residual weight of 20 ± 2% was obtained at 800 °C which is in agreement with the ash content reported for acemannan [50].

#### 3.1.4. TPC and Antioxidant Activity

The TPC value found for AVR was 201.053 ± 0.002 mg GAE g^−1^ sample (dry weight) whereas an IC_50_ value of 0.726 ± 0.009 mg extract mL^−1^ was obtained by the DPPH method. These results are in accordance with reported literature for the same Aloe Vera gel variety [27]. The obtained high TPC and low IC_50_ values could be related to the presence of phenolic compounds in AVR, mainly chlorogenic acid and caffeic acid, which possess redox properties and a high capacity in reducing free radicals. Moreover, the presence of many other antioxidants in AV has been demonstrated, including ascorbic acid, α-tocopherol and carotenoids, among others [13,14]. This fact also explains the high values obtained by the FRAP and ABTS methods (35.16 ± 0.04 µg GAE and 25.4 ± 0.1 µg GAE per gram of dried extract, respectively) underlining the potential of AV as an antioxidant additive and the scavenging of free radicals as an important mechanism of protection in Aloe gel [21,27,29].

### 3.2. Characterization of Edible Films

#### 3.2.1. Visual Appearance, Thickness and Transparency

**Figure 2:**
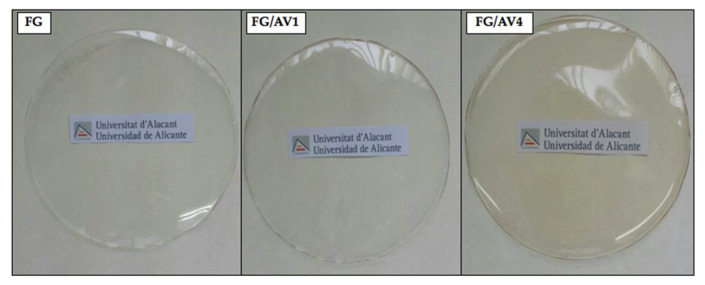
Visual appearance of FG, FG/AV1 and FG/AV4 edible films.

The visual appearance of the obtained films is shown in Figure 2. In general, all films showed a high visual transparency and homogeneity, which are desirable properties for food packaging applications. However, a slight yellowish coloration was observed at high AV loadings (FG/AV4) [20]. No significant differences were obtained regarding thickness values for FG (104 ± 9 µm), FG/AV1 (110 ± 20 µm) and FG/AV4 (110 ± 10 µm) suggesting that AV addition did not substantially affect the film formation process [21]. However, the addition of AV to FG induced some changes in films transparency. Indeed, transparency values of films to ultraviolet radiation (280 nm/mm) increased from 67 ± 3 for FG to 74 ± 2 for FG/AV1 and 101 ± 3 for FG/AV4, whereas transparency values to visible radiation (%) also increased with AV content from 37 ± 2 for FG to 42 ± 2 for FG/AV1 and 60 ± 2 for FG/AV4. This effect could be related to the crosslinking of polysaccharides and antioxidants present in AV with FG proteins or glycerol (plasticizer) in the film forming process [51]. In general terms, the neat visual appearance and high transparency of the studied edible films were adequate for food packaging applications in all cases, since the addition of AV to form FG/AV1 and FG/AV4 does not affect in the visual appareance of the packed product.

#### 3.2.2. Morphological Characterization by SEM

**Figure 3:**
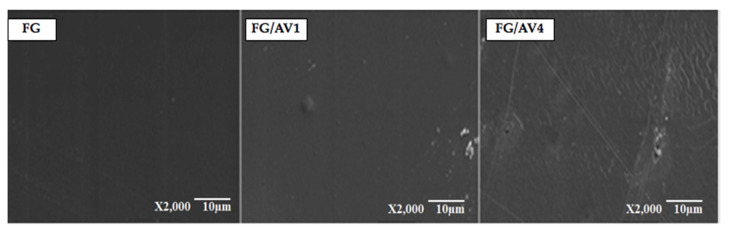
Surface micrographs obtained for FG, FG/AV1 and FG/AV4 edible films by SEM (2000×).

Figure 3 shows the surface micrographs obtained for each edible film. FG showed a smooth and continuous surface whereas a slightly rougher surface was observed in FG/AV films as AV content increased. Similar results were observed for alginate films added with Aloe Vera gel extract [20]. According to different authors, roughness could be related to chemical interactions through the formation of covalent and non-covalent bonds between gelatin, glycerol and AV [33]. Nevertheless, no component segregation or visual discontinuities were observed on the surface of FG/AV films with the addition of AV, which is a positive result for potential packaging applications.

#### 3.2.3. Structural Characterization by ATR-FTIR

**Figure 4:**
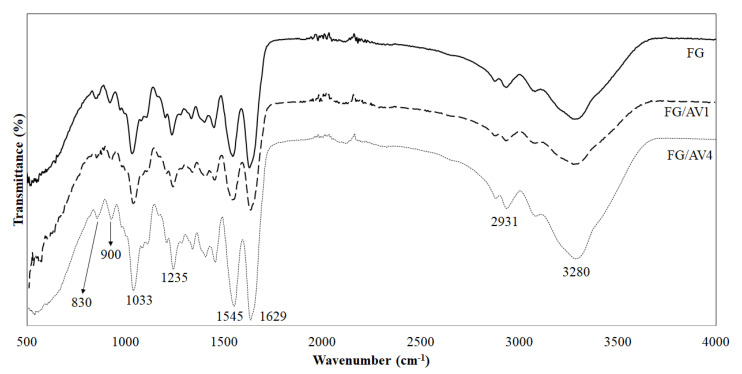
Average ATR-FTIR spectra obtained for FG, FG/AV1 and FG/AV4 edible films.

Figure 4 shows the averaged ATR-FTIR spectra obtained for all edible films. Six characteristic bands related to FG matrix were observed. A broad absorption band around 3280 cm^−1^ was correlated to the stretching vibration of hydroxyl groups (O-H) whereas the intense peak observed around 2931 cm^−1^ was attributed to the stretching vibrations of aliphatic C-H in CH_3_ groups [33]. The bands at approximately 1629, 1545, and 1235 cm^−1^ were assigned to the amide-I (C=O stretching/hydrogen bonding coupled with COO), amide-II (N-H bending vibration of N-H groups and stretching vibrations of C-N groups) and amide-III (C-N and N-H stretching groups of bound amide or vibrations of CH_2_ groups of glycine), respectively [5,6]. The peak at approximately 1033 cm^−1^ was assigned to the C-O stretching of glycerol, used as plasticizer [33].

Some small differences were observed among ATR-FTIR spectra of the studied formulations which occurred only in limited wavelength regions. In these cases, statistical methods allow the extraction of analytical information from the full spectra. Table 2 shows the maximum wavenumber and transmittance values of some specific bands observed in the spectra for all samples. Generally, as AV content increased, lower transmittance values were obtained for all bands. According to the manufacturer specifications, the FG used in this work is composed of different amino acids with hydrophobic (glycine, proline, alanine, phenylalanine, leucine, methionine and valine), hydrophilic (hydroxyproline, serine and threonine), positively charged (arginine and lysine) and negatively charged (glutamic acid and aspartic acid) nature. Thus, interactions between FG and AV through hydrogen bonding and Van der Waals forces between amino acids (free -OH group, -COOH end groups and -NH_2_), polysaccharides, antioxidants and glycerol (plasticizer) might likely occur with a decrease on transmittance values [8,33]. In this line, some authors have observed a broadening effect in OH and NH bands with the addition of chitosan to a gelatin matrix suggesting hydrogen bonding interactions, which were related to a good incorporation of AV into the polymer matrix [52]. Also, Hosseini et al. [33] reported an increase in some characteristic bands of biocomposite films based on fish gelatin and chitosan with the addition of Origanum vulgare L. essential oil to the polymer matrix, and this effect was presumably attributed to the interactions established between the polymer matrix and the essential oil.

Finally, as AV content increased, a significant shift (*p* < 0.05) to higher wavenumbers values in bands assigned to the presence of glucose and mannose monosaccharides was observed among samples, resulting in values of 894 ± 7 cm^−1^ and 817 ± 6 cm^−1^ for FG to 913 ± 2 cm^−1^ and 835 ± 4 cm^−1^ for FG/AV4, respectively. These shifts could be correlated to conformational changes on the polymer matrix with AV addition and a good molecular affinity between FG and AV [20,33].

#### 3.2.4. Thermal Characterization

**Figure 5:**
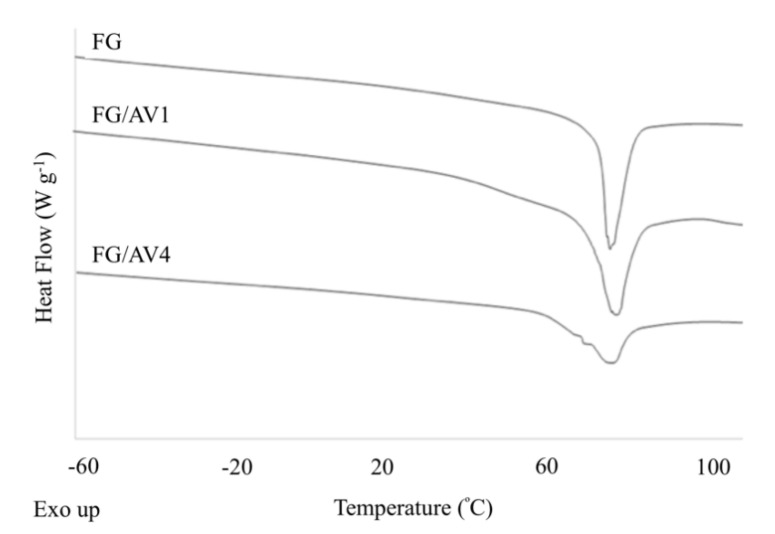
DSC thermograms obtained for FG and FG/AV edible films.

Figure 5 shows the DSC curves obtained for all edible films. One endothermic peak was observed in all films which was related to the disruption of the crystalline or ordered phase due to changes from the initial stabilized native structure of the fish protein to a denatured state as a consequence of heating [5]. Also, glass transition temperatures (T_g_) ranging from 16 to 40 °C were observed. T_g_ is normally correlated to the segmental motion of polymer molecules in the amorphous phase. As it can be seen in Table 2, FG showed a denaturation temperature (T_d_) and enthalpy (ΔH_d_) of 80 ± 4 °C and 26 ± 2 J g^−1^, respectively, and a T_g_ around 22 ± 6 °C. In general, as AV content increased, the thermal parameters related to the denaturation process tend to decrease whereas T_g_ values increased due to the disruption of the FG structure with partial destabilization of the intramolecular hydrogen bonds inside the protein matrix and the formation of intermolecular bonds with certain crosslinking between proteins, polysaccharides and antioxidant compounds [5,33,46]. Some authors have suggested the formation of hydrogen bonds between hydrophilic AV compounds and the hydrophilic gelatin matrix, as well as the crosslinking between the gelatin molecules and phenolic compounds present in AV. Also, hydrophobic interactions could be achieved between the hydrophobic groups of polyphenols and the hydrophobic region of FG [34]. A similar behaviour was observed in tilapia skin gelatin-based films added with 0.05% of an ethanolic coconut husk extract showing a T_g_ value of 54 °C in contrast to 47 °C for the control film without the extract. In this case, the authors stated that interactions between the gelatin molecules and the phenolic compounds present in the extract were responsible for increasing the T_g_ values [34].

**Figure 6:**
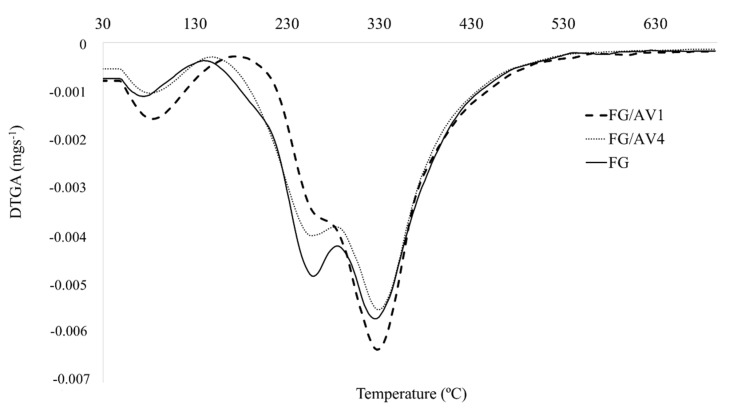
Derivative thermogravimetric (DTG) curves obtained for FG and FG/AV edible films.

DTG curves of FG and FG/AV films are shown in Figure 6. The maximum degradation temperatures of the three observed degradation steps (T_max_), volatiles loss (%) and residual weight at 700 °C of all films are shown in Table 3. A first stage weight loss (8–9%) was observed at a maximum degradation temperature ranging from 81 to 85 °C, which was associated to the loss of volatiles as well as free and bound water adsorbed in the film [9,53]. The second stage weight loss appeared at a maximum degradation temperature (T_max2_) ranging from 259 to 263 °C. This transition was correlated to the degradation or decomposition of low molecular weight protein fractions and glycerol present in the film matrix [6]. A final third stage of weight loss was observed for all films at a maximum degradation temperature (T_max3_) between 328–330 °C which was possibly caused by the loss or decomposition of larger-size or highly interacted proteins and high temperature stable components present in the film matrix [54]. A non-volatile residual material was found at 700 °C (residual weight) with values between 15–21%. According to the TGA results obtained for the studied films it could be concluded that the addition of AV to the FG matrix at the studied concentrations (1 and 4 wt.%) did not significantly affect the thermal stability of the obtained FG/AV edible films (Table 3) [5,6]. These results also suggested that the AV addition did not reduce the heat resistance of the gelatin-based films [53].

The obtained FG/AV films improved their thermo-oxidative resistance compared to the control FG film according to the obtained OOT and T_ini_ values. These parameters increased with AV content with values of 216 ± 2 °C and 223 ± 6 °C for FG, 221 ± 1 °C and 230 ± 2 °C for FG/AV1 and 228 ± 1 °C and 237 ± 4 °C for FG/AV4, respectively. This behaviour was associated with the antioxidant effect induced by the presence of bioactive compounds, such as polyphenols, quinones and polysaccharides, in AV, in agreement with the TPC and antioxidant activity results obtained in this work [11,12,19,21]. Some authors have also reported a slight increase in the degradation temperature of alginate-based blends with the addition of Aloe Vera due to the formation of polymer-polymer interactions [20]. The OOT and T_ini_ results obtained might also support the T_g_ values obtained by DSC as well as the statistical differences found by FTIR, suggesting a good incorporation of AV into the FG matrix.

#### 3.2.5. Mechanical and Barrier Properties

Table 4 summarizes the results obtained for mechanical and barrier properties in all edible films. The hydrophilic character of AV significantly increased (*p* < 0.05) the solubility of the developed films, as expected [26,46].

Fish gelatin is hydrophilic in nature, due to the presence of polar amino acids and large number of hydroxyl groups (−OH). OTR results showed low values for all films, which are in accordance with the reported results for fish gelatin and chitosan polymeric films by Evranos et al. [55]. Lower WVP values for fish gelatin films than those obtained in this study were reported for tilapia skin gelatin [9,34,53]. These differences can be due to the variation in film thicknesses, which were around 0.050 mm in the reported work, while it was 0.104 mm in average in the present study. All films displayed low solubility values, around 40%, similar to those reported by Jeya Shakila et al. [56] and Hosseini et al. [33] in composite films obtained from fish gelatin and chitosan. All the prepared films maintained their integrity after incubation in water for 24 h. This resistance to moisture is a substantial characteristic of edible films for use in food protection where moisture is critical in the deterioration of packaged food [33].

FG/AV4 film showed a slight but not significant increase in the elastic modulus and WVP (*p* > 0.05) values, suggesting that mechanical and barrier properties were not significantly affected by the addition of AV up to 4 wt.%. This behaviour is in accordance with the results by Sui Chin, Lyn and Hanani [21] who reported no significant effects in barrier properties, tensile strength and elongation at break values of FG-based films after the addition of AV contents of 5 and 9 wt.%. Adequate mechanical strength of an edible biopolymer film is necessary to protect the integrity of materials for food packaging throughout distribution. Although the higher T_g_ values, already discussed in Section 3.2.4, might suggest a certain degree of stiffening, this effect seems to be not enough to be reflected in a significant change in the mechanical behaviour of the obtained FG/AV films. Thus, satisfactory mechanical and barrier properties were still obtained since the AV contents studied in this work do not significantly affect the naturally acceptable properties of FG, and higher concentrations might be needed to be studied in order to achieve a reinforcement effect [34].

#### 3.2.6. Antimicrobial Activity

**Figure 7:**
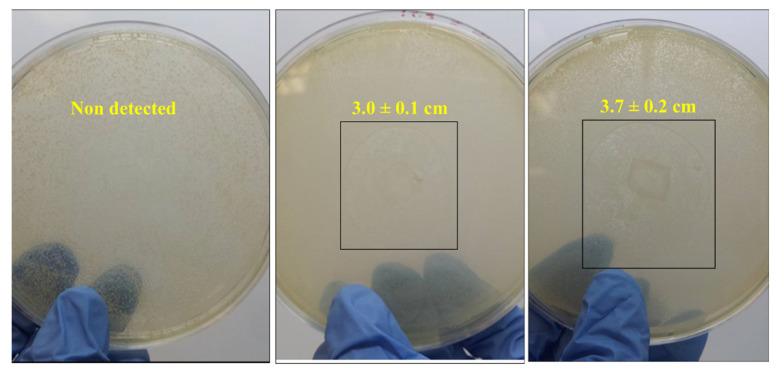
Antimicrobial activity of FG/AV films in contact with *S. aureus* bacterial strain.

Significant differences in the antimicrobial performance of FG/AV films in contact with the two studied bacterial strains were observed. For *S. aureus* (Figure 7), inhibition zones of 3.0 ± 0.1 cm and 3.7 ± 0.2 cm were found for FG/AV1 and FG/AV4, respectively, in contrast to the control FG film which did not show any inhibition zone, as expected. This antibacterial activity could be attributed to the presence of polyphenols and other antioxidants in AV with ability to produce the precipitation of cell membrane proteins or having non-specific interactions with them [18,57,58], in agreement with the high TPC and antioxidant activity values obtained in this work. On the other hand, the acemannan present in AV has been reported to be also responsible for antibacterial activity [2]. In this sense, the action of AV polysaccharides against bacterial activity has been well reported, including the stimulation of phagocytic leucocytes and T cells as well as the induction of nitric oxide production to destroy bacteria [13,59]. Finally, some authors have suggested that an increase in the solubility of films could influence the release of antimicrobial components resulting in higher releasing rates [24]. In this work, the addition of AV significantly (*p* < 0.05) increased the solubility of films from 31 ± 1% for FG to 44 ± 1% for FG/AV4 (Table 3). Thus, it was expected that FG/AV4, with higher TPC, antioxidant activity and solubility values, would show the highest antimicrobial effect against *S. aureus*. However, no inhibition zones were observed for S. enterica in any of the studied films which was attributed to the additional external lipopolysaccharide membrane present in this bacterium which contributes to a higher resistance to disintegration [35]. A higher action against gram positive bacteria compared to gram negative microorganisms was also reported in Aloe Vera gel-based edible coatings to reduce the ripening process of sweet cherry [2]. According to some authors, the Aloe Vera gel extract could be used as an antifungal agent to prevent postharvest fungal diseases as it has been recently proved in papaya fruit [11,60].

## 4. Conclusions

The addition of Aloe Vera gel at 1 and 4 wt.% into a FG matrix was successfully performed to obtain active edible films with antimicrobial properties against *S. aureus*. The linkage analysis of AV underlined the presence of acemannan as active polysaccharide. The carbohydrate composition of AV evidences that no extraction or purification steps are actually necessary to obtain a gel. AV also showed high antioxidant performance according to the obtained TPC and antioxidant activity results which directly improved the thermo-oxidative performance of the FG/AV films. The AV incorporation did not significantly affect mechanical and barrier (OTR, WVP) properties of the resulting films. However, some structural changes were observed by FTIR and DSC with AV addition due to the presence of intermolecular interactions that increased the hydrophilicity and solubility of the FG/AV films. A high visual transparency and homogeneity was observed in the developed films, which are important and desirable properties for films intended for food packaging applications. In conclusion, this work underlines the potential application of the developed antibacterial edible films based on FG and AV inner gel, in particular at 4 wt.% AV concentration, to increase the postharvest quality of packaged food, such as fruits and vegetables with low water content (nuts, potato snacks, bean sprouts, avocado, etc.), reducing the use of synthetic additives and contributing to the circular economy concept by reducing food wastes. Further work will be needed to evaluate the antimicrobial properties of these films directly applied to food products as well as to study the sensorial behaviour of the packed food to improve the functional properties of the obtained edible films. Moreover, the addition of higher AV contents could also be studied to evaluate a possible enhancement in antimicrobial properties against different food microorganisms and getting new applications in the food packaging sector.

## Figures and Tables

**Table 1:** Carbohydrate linkage analysis of AV (mean ± SD, *n* = 3).

Linkage	Relative Abundance (%mol)
t-Ara*f*	0.50 ± 0.08
5-Ara*f*	0.09 ± 0.03
**Total Ara**	**0.59** ± **0.12**
t-Xyl*p*	0.09 ± 0.01
2-Xyl*p*/4-Xyl*p* *	1.01 ± 0.15
2,4-Xyl*p*	0.17 ± 0.05
**Total Xyl**	**1.26** ± **0.22**
t-Glc*p*	1.00 ± 0.24
2-Glc*p*	0.23 ± 0.08
3-Glc*p*	0.13 ± 0.02
4-Glc*p*	10.80 ± 0.50
4,6-Glc*p*	0.29 ± 0.03
**Total Glc**	**12.45** ± **0.87**
t-Man*p*	0.64 ± 0.10
2-Man*p*	0.20 ± 0.02
4-Man*p*	82.16 ± 0.90
3,4-Man*p*	0.08 ± 0.03
2,4-Man*p*	0.20 ± 0.09
4,6-Man*p*	0.28 ± 0.05
**Total Man**	**83.56** ± **1.17**
t-Gal*p*	0.47 ± 0.08
3-Gal*p*	0.75 ± 0.12
6-Gal*p*	0.26 ± 0.03
3,6-Gal*p*	1.06 ± 0.34
**Total Gal**	**2.54** ± **0.58**
t-Gal*p*A	0.03 ± 0.01
4-Gal*p*A	0.26 ± 0.15
**Total GalA**	**0.29** ± **0.60**
t-Glc*p*A	0.01 ± 0.01
4-Glc*p*A	0.02 ± 0.01
**Total GlcA**	**0.03** ± **0.02**

Ara (Arabinose), Xyl (Xylose), Glc (Glucose), Man (Mannose), Gal (Galactose), GalA (Galacturonic acid) and GlcA (Glucuronic acid). Only traces of Fucose and no detectable Rhamnose were present in the samples. * Peaks co-elute.

**Table 2:** FTIR parameters obtained for FG and FG/AV edible films (mean ± SD, *n* = 3).

Wavenumber (cm^−1^)			Transmittance (%)		
FG	FG/AV1	FG/AV4	FG	FG/AV1	FG/AV4
3281 ± 12 ^a^	3273 ± 9 ^a^	3283 ± 1 ^a^	79 ± 11 ^a^	78 ± 9 ^a^	55 ± 2 ^b^
2932 ± 2 ^a^	2932 ± 1 ^a^	2932 ± 1 ^a^	88 ± 7 ^a^	86 ± 5 ^a,b^	73 ± 1 ^b^
1630 ± 1 ^a^	1629 ± 1 ^a^	1631 ± 1 ^a^	67 ± 17 ^a^	62 ± 14 ^a,b^	31 ± 2 ^b^
1546 ± 1 ^a^	1542 ± 5 ^a^	1545 ± 1 ^a^	70 ± 15 ^a^	66 ± 12 ^a^	36 ± 2 ^b^
1237 ± 1 ^a^	1236 ± 1 ^a^	1237 ± 1 ^a^	76 ± 12 ^a^	72 ± 10 ^a,b^	49 ± 1 ^b^
1034 ± 1 ^a^	1033 ± 1 ^a^	1034 ± 1 ^a^	72 ± 15 ^a^	67 ± 11 ^a,b^	41 ± 1 ^b^
894 ± 7 ^a^	898 ± 3 ^a^	913 ± 2 ^b^	92 ± 1 ^a^	78 ± 2 ^b^	71 ± 1 ^c^
817 ± 6 ^a^	829 ± 2 ^b^	835 ± 4 ^b^	90 ± 2 ^a^	77 ± 1 ^b^	72 ± 1 ^c^

Different superscripts within the same line for each FTIR parameter indicate statistically significant different values (*p* < 0.05).

**Table 3:** Thermal properties obtained for edible films by DSC and TGA (mean ± SD, *n* = 3).

	Thermal Parameter	FG	FG/AV1	FG/AV4
DSC parameter	ΔH_d_ (J g^−1^)	26 ± 2 ^a^	27 ± 4 ^a^	15 ± 4 ^b^
	T_d_ (°C)	80 ± 4 ^a^	83 ± 1 ^a^	71 ± 5 ^a^
	T_g_ (°C)	22 ± 6 ^a^	34 ± 5 ^b^	38 ± 2 ^b^
TGA parameter	Volatiles loss (%)	8 ± 1 ^a^	9± 1 ^a^	9 ± 2 ^a^
	T_max1_ (°C)	81 ± 2 ^a^	85 ± 1 ^a^	83 ± 3 ^a^
	T_max2_ (°C)	259 ± 1 ^a^	259 ± 2 ^a^	263 ± 5 ^a^
	T_max3_ (°C)	330 ± 3 ^a^	329 ± 2 ^a^	328 ± 2 ^a^
	Residual weight (700 °C, %)	15 ± 6 ^a^	21 ± 1 ^a^	21 ± 1 ^a^

Different superscripts within the same raw and thermal parameter indicate statistically significant different values (*p* < 0.05).

**Table 4:** Mechanical (*n* = 5) and barrier properties (*n* = 3) obtained for FG and FG/AV edible films (mean ± SD).

Property	FG	FG/AV1	FG/AV4
Elastic modulus (MPa)	1150 ± 130 ^a^	1100 ± 200 ^a^	1400 ± 300 ^a^
Elongation at break (%)	9 ± 3 ^a^	6 ± 3 ^a^	8 ± 3 ^a^
Tensile strength (MPa)	36 ± 2 ^a^	40 ± 10 ^a^	38 ± 8 ^a^
OTR.e (cm^3^ mm m^−2^ day)	0.41 ± 0.08 ^a^	0.45 ± 0.03 ^a^	0.45 ± 0.08 ^a^
WVP × 10^−11^ (kg m Pa^−1^ s^−1^ m^−2^)	4.2 ± 1.2 ^a^	4.0 ± 0.4 ^a^	4.8 ± 0.6 ^a^
Solubility (%)	31 ± 1 ^a^	34 ± 2 ^a^	44 ± 1 ^b^

Different superscripts within the same line and parameter indicate statistically significant different values (*p* < 0.05).

## Supplementary Materials

The following files are available online at https://www.mdpi.com/2304-8158/9/9/1248/s1, Figure S1: sugar composition of AV after a two-step sulphuric hydrolysis and TFA hydrolysis, Figure S2: Thermal characterization of AV by DSC, Figure S3: DTG curve obtained of AV by TGA.
