# Supplementary figures and images for: Physicochemical and Functional Properties of Active Fish Gelatin-Based Edible Films Added with Aloe Vera Gel
Source: Foods. 2020 Sep 7;9(9):1248. doi: 10.3390/foods9091248 (PMC7555046; doi:10.3390/foods9091248)

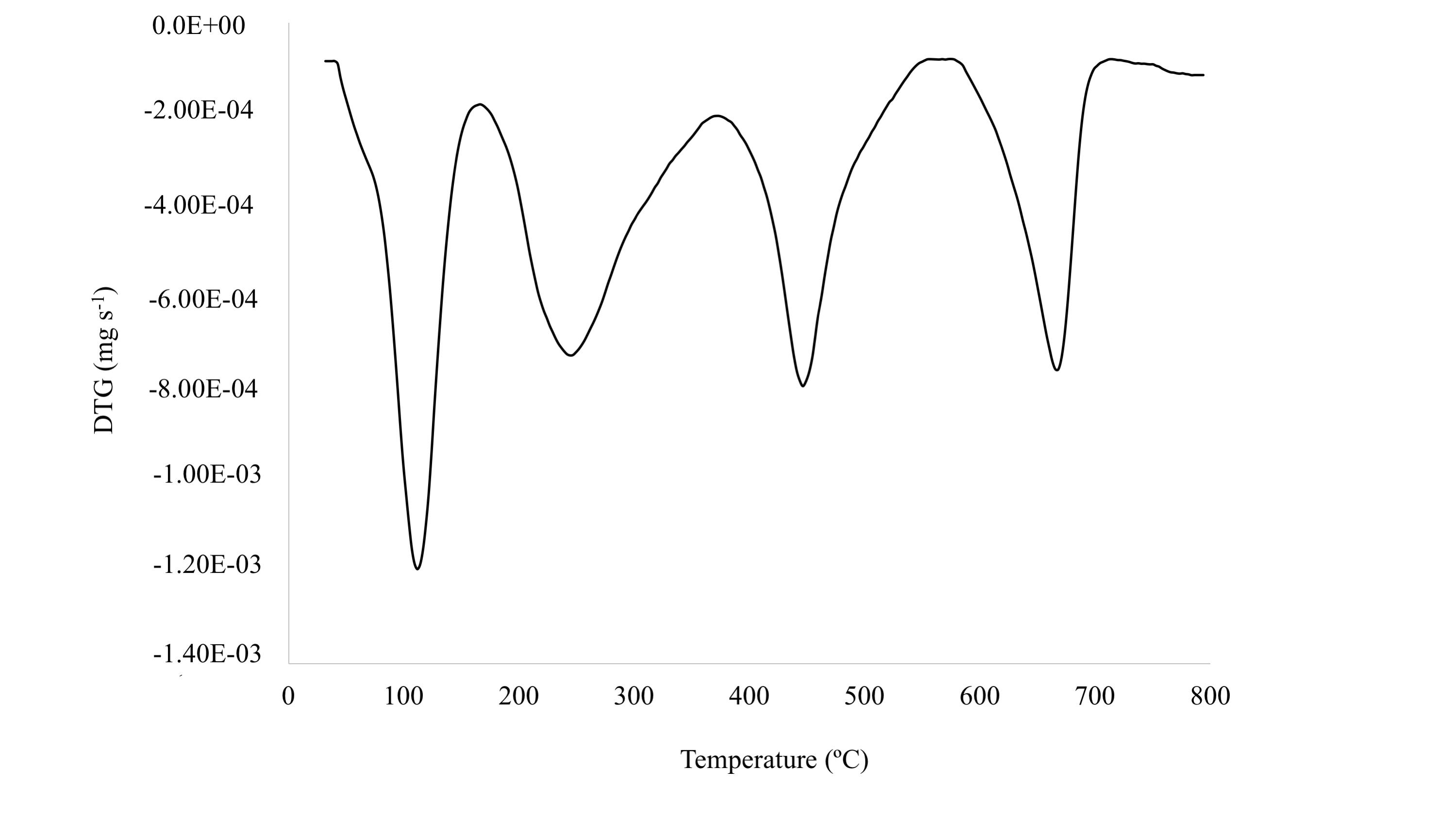

Supplement: Supplementary file 1 [file foods-09-01248-s001.zip › Supplementary Files/Figure S3.tiff]

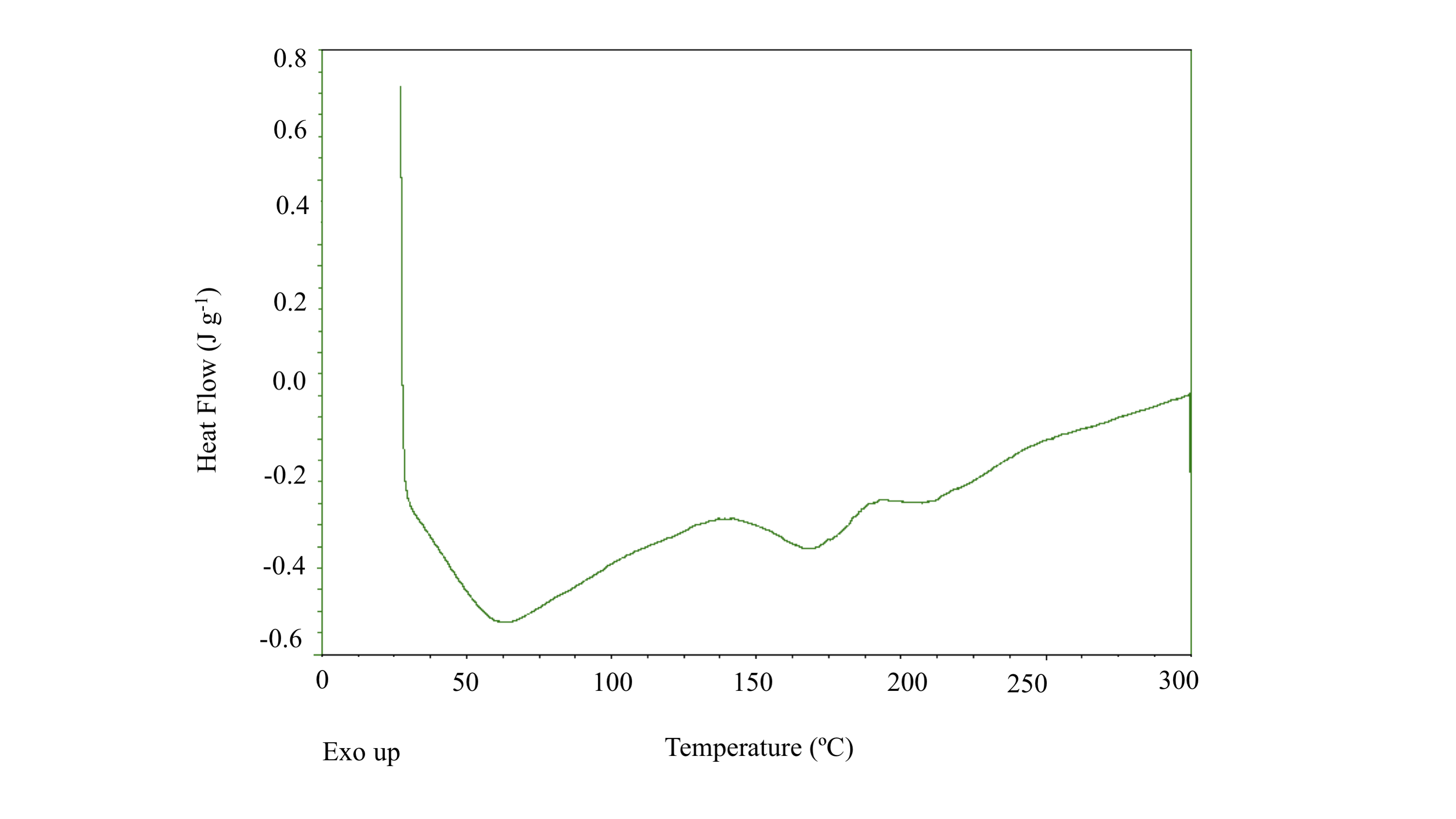

Supplement: Supplementary file 1 [file foods-09-01248-s001.zip › Supplementary Files/Figure S2.tiff]

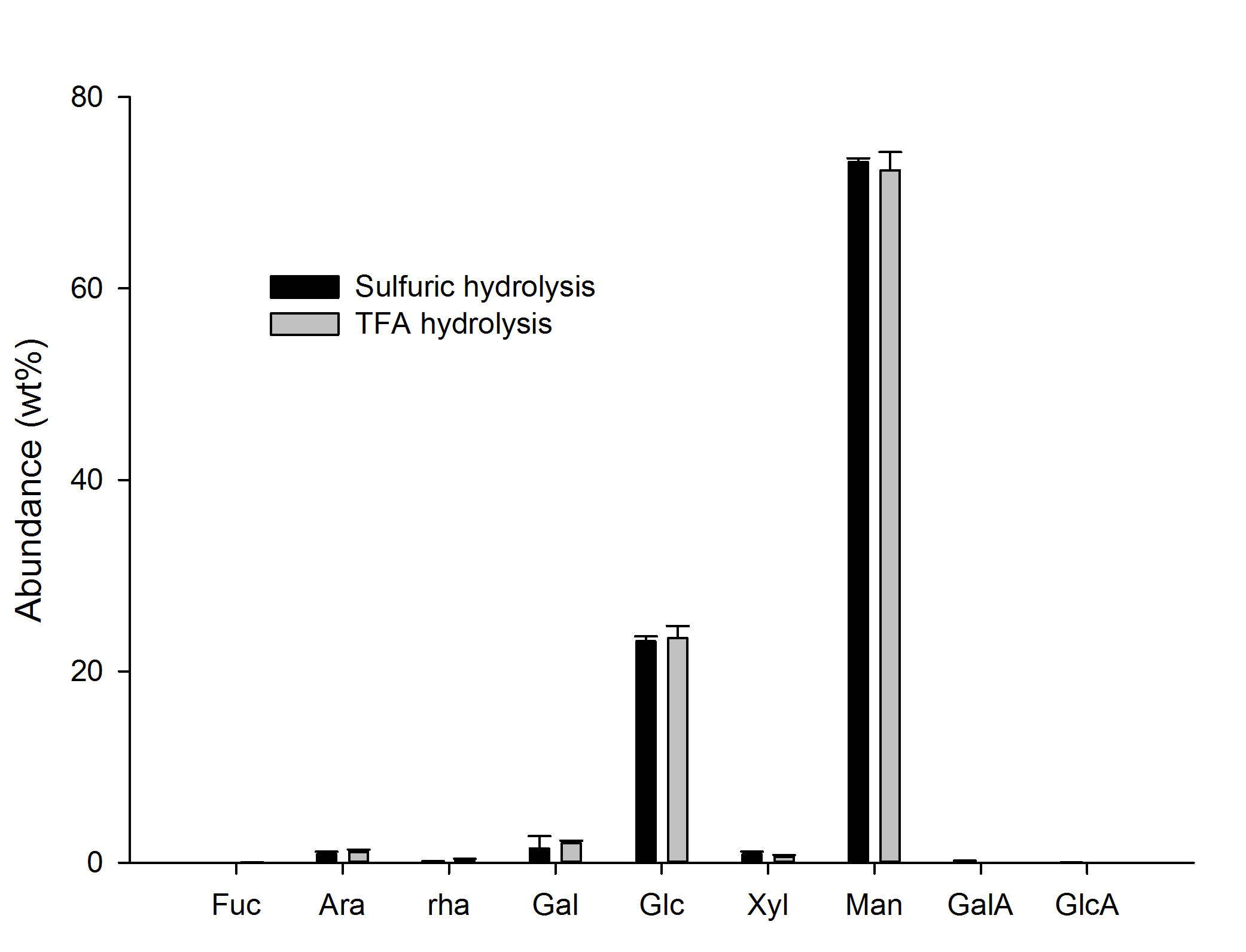

Supplement: Supplementary file 1 [file foods-09-01248-s001.zip › Supplementary Files/Figure S1.JPG]
